# Encapsulation of berberine decorated ZnO nano-colloids into injectable hydrogel using for diabetic wound healing

**DOI:** 10.3389/fchem.2022.964662

**Published:** 2022-08-09

**Authors:** Xuechen Yin, Xiangyi Fan, Zipeng Zhou, Qi Li

**Affiliations:** ^1^ College of Basic Medicine, Jinzhou Medical University, Jinzhou, China; ^2^ Department of Otolaryngology-Head and Neck Surgery, First Affiliated Hospital of Jinzhou Medical University, Jinzhou, China; ^3^ Department of Orthopedics, First Affiliated Hospital of Jinzhou Medical University, Jinzhou, China; ^4^ First Affiliated Hospital of Jinzhou Medical University, Jinzhou, China

**Keywords:** ZnO, nano-colloids, berberine, oxidative stress, inflammatory, wound healing

## Abstract

Chronic wound healing in diabetic patients had been considered a major clinical challenge, so there was an urgent need to establish more effective treatment methods. In this study, we prepared berberine-modified ZnO nano-colloids hydrogel (ZnO-Ber/H) and evaluated its wound healing performance in a diabetic rat. The prepared ZnO-Ber/H had excellent moisturizing, anti-inflammatory and anti-oxidative stress abilities. *In vitro*, ZnO-Ber/H could effectively up-regulate antioxidant stress factors (Nrf2, HO-1, NQO1) by 4.65-fold, 2.49-fold, 2.56-fold, respectively. *In vivo* experiments have shown that ZnO-Ber/H could effectively improve the wound healing rate (92.9%) after 15 days of treatment. Meanwhile, the ability of anti-oxidative stress had also been verified *in vivo*. ZnO-Ber/H down-regulated inflammatory factor (TNF-α, IL-1β, and IL-6) by 72.8%, 55% and 71% respectively, up-regulated vascular related factors VEGF and CD31 by 3.9-fold and 3.2-fold by Western blot. At the same time, ZnO-Ber/H could promote the expression of EGFR and FGFR, thereby affecting the generation of new epithelial tissue. Based on extensive characterization and biological evaluation, ZnO-Ber/H was expected to be a potential candidate for promoting diabetic wound healing.

## Introduction

Wound healing of diabetic patients was delayed due to various complex reasons (Ren et al., 2022; Zhao et al., 2022). In these patients, hyperglycemia attenuated the interaction between various growth factors and target sites around the wound, as well as the lack of angiogenesis and re-epithelialization. Meanwhile, severe inflammatory infiltration was another harmful factor (Samadian et al., 2020). Relevant studies indicated that oxidative stress could further damage and retard the process of wound healing. In the meanwhile, the increase of oxidative stress was a common feature in the development of diabetes (Sen and Roy, 2008; Lan et al., 2013; Lin et al., 2020; Deng et al., 2021). Although tremendous breakthroughs had been made in the past long time, we still need to prepare multifunctional anti-inflammatory and anti-oxidant composite materials to reverse the development of chronic wounds in diabetic patients.

An excellent wound dressing should prevent dehydration, keep the wound moist, allow gas penetration, ensure isolation from the invasion of external pathogens. By maintaining the wound humid, wound dressing could help prevent bacterial infection and promote effective healing (Zhang et al., 2021; Huang et al., 2022; Xu et al., 2022). Polyvinyl alcohol/sodium alginate hydrogel system, as a commonly used hydrogel sustained release system, had been widely applied due to its non-toxic, biocompatible, high hydrophilic, chemical resistance and mechanical properties (Mohammadi et al., 2019; Chen et al., 2020; Zhang et al., 2020; Ding et al., 2021). ZnO had high specific surface area, the ability to produce reactive oxygen species and strong surface chemical reaction, which met all the necessary requirements for the full treatment of bacterial infection. In addition, recent research evidence indicated that ZnO with appropriate concentration could show antibacterial ability without any side effects on human cells (Manzoor et al., 2016; Guy et al., 2019; Alavi and Nokhodchi, 2020; Dodero et al., 2020). ZnO with smaller size could generate more reactive oxygen species, have higher defect concentration, and therefore have stronger antibacterial ability. The presence of antioxidant factors in cells resists the reactive oxygen species produced by zinc oxide (Guy et al., 2019). At the same time, it was a fact that zinc deficiency delays wound healing in clinic. Several clinical and experimental studies on ZnO were carried out (Lansdown et al., 2007). These results indicated that the treatment based on local application of ZnO had some profits in re-epithelialization, infection and ulcer. Berberine (Ber) had extensive characteristic, like anti-oxidative stress, antibacterial, antifungal and anti-inflammatory (Ahmed et al., 2015). Therefore, whether it is possible to match the strong anti-oxidative stress ability of berberine with the ability of zinc oxide to prepare materials more suitable for promoting wound healing.

**SCHEME 1:**
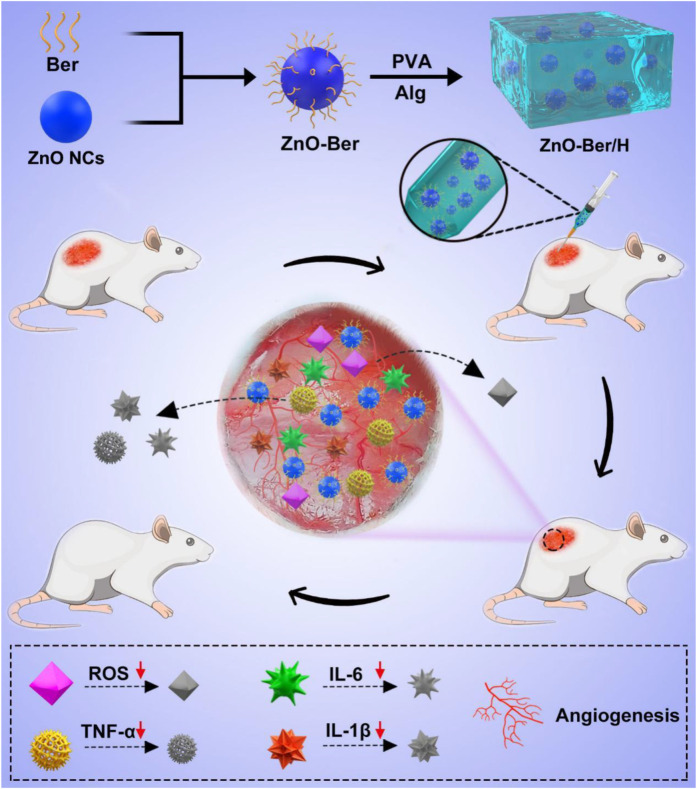
Schematic synthetic process of ZnO-Ber/H multifunctional composite hydrogel and its application to wound in diabetic rat.

In this work, to achieve these goals, we combined ZnO nano-colloids (ZnO NCs) and berberine to upgrade it into a hydrogel as an effective tool for the repair and regeneration of skin lesions in diabetic rats (Scheme 1). Because of the larger specific surface area of ZnO, berberine molecules could be enriched on its surface. Subsequently, we uniformly mixed it in the hydrogel system to prepare a new type of composite material that had strong anti-inflammatory and anti-oxidative stress and accelerated the healing of diabetic wounds. ZnO-Ber/H was coated on the wound of rats to reduce the level of oxidative stress and inflammation related factors IL-1B, IL-6 and TNF- α. After that, the wound would form a microenvironment in the wound that is conducive to the vascularization of the body. We used NIH-3T3 and HaCaT cells to study the potential of ZnO-Ber/H for proliferation and migration at suitable concentration. After that, we found that ZnO-Ber/H could not only promote the expression of EGFR and FGFR, but also enhance the expression of anti-oxidative stress-related molecules and inhibit inflammation-related factors through AKT signaling pathway *in vivo*.

## Materials and methods

### Regents and preparation process

Deionized aqueous solution (5 ml) containing zinc sulfate (0.01 M). Then, sodium hydroxide (0.12 M), ethanol (20 ml) and linoleic acid (4 ml) were mixed uniformly. The zinc sulfate solution was mixed with the above solution, sonicated and heated at 120°C. After 12 h, the solution was cooled and the product was collected by centrifugation and washing with water. Finally, we got ZnO NCs. All the above reagents were purchased from Macklin.

After obtaining ZnO NCs (50 mg), 20 ml of an aqueous solution containing berberine (50 mg) was added, and the pH was adjusted with buffer. Then, the solution was stirred at 40°C for 3 h. The solution was finally filtered and the precipitate was collected, washed and freeze-dried to get ZnO-Ber powder. All the above reagents were purchased from Macklin.

Then, 10 ml polyvinyl alcohol solution [8% (w/v)] were mixed and stirred with sodium alginate (0.1 g) powder in a 50 °C water bath. ZnO-Ber powder was then added for 2 h to gelate, and the product (ZnO-Ber/H) was allowed to stand at 37 °C for 3 h. All the above reagents were purchased from Macklin.

### Characterization

The morphologies and sizes of ZnO-Ber was characterized by transmission electron microscopy (TEM, Zeiss, Germany). The chemical structures of all groups were tested by Fourier transform infrared (FT-IR) spectroscopy (Thermo, United States). UV-vis data were obtained by UV–vis spectrophotometer (Scinco 4100). Dynamic light scattering (DLS) data were recorded on a zetasizer (Malvern, Nano ZS90, Worcestershire, U.K.). Thermogravimetric analysis by differential scanning calorimetry (DSC, Mettler Toledo, TGA/DSC1/1100, Switzerland).

### Cell culture

Human immortalized keratinocytes (HaCaT) and Mouse embryonic fibroblast cells (NIH-3T3) in DMEM medium (Gibco, United States) containing 1% penicillin-streptomycin (Gibco, United States) and 10% fetal bovine serum (FBS, Gibco) at 37°C (5% CO_2_) in an incubator.

### Cell viability assay

The CCK-8 assay and live and dead cell staining kit were used for assessing the biocompatibility of the B/H and ZnO-Ber/H. NIH-3T3 cells were mixed with live & dead staining solution for 15 min. The CCK-8 assay was used for assessing the proliferation of NIH-3T3 and HaCaT cells. The cells were cultured in 96-well plates for 24 h, then, B/H and ZnO-Ber/H were added for 24 h. After that, 10 μl of CCK-8 was supplemented each 100 μl of medium. Lastly, Optical density values (450 nm) were analyzed by microplate reader.

### Scratch assay

NIH-3T3 were cultured in 6-well plates at 37°C (5% CO_2_). When the cell density reached the standard, a line was drawn on the bottom of the plates with the tip of a sterile pipette. Then NIH-3T3 were cultured with B/H and ZnO-Ber/H separately in incubator. Images were obtained at 0 and 12 h posttreatment by an inverted microscope.

### Detection of intracellular ROS levels

DCFH-DA assay was employed for ROS measurement (Solarbio, Beijing, China). In this work, we pretreated fibroblasts with LPS (1 μg/ml) for 1 h, after that, the cells were treated with B/H (1 mg/ml), ZnO-Ber/H (1 mg/ml) for 4 h. Add the diluted DCFH-DA (10 μM/L) to each group of dishes, and maintained it in an incubator for 20 min, washed three times with FBS-free medium. Results were observed by a confocal fluorescence microscope. SOD, LDH, MDA, and NO test kits were purchased from Beyotime company.

### Animal experiment

Sprague-Dawley (SD) rats (about 200 g) were purchased from Liaoning Changsheng biotechnology Company. After dissolving STZ (80 mg/kg) with citrate buffer (0.1 M, pH 4.5), the rats were injected intraperitoneally to establish a diabetic animal model. The blood glucose levels were examined with glucometer, rats with glucose level above 19.2 mM were identified as successful diabetic models. After that, the rats were anesthetized with isoflurane. Next, a round full thickness excision wound of 1.5 × 1.5 cm^2^ was produced on each side of each rat’s back. The Hydrogel, B/H, and ZnO-Ber/H were applied to the wound every day. Finally, the wound area was measured every 5 days (*n* = 4 for each group).

### Tissue histology

Fifteen days after treatment, all the animals were euthanized and the skin were collected, then fixed in 4 wt% paraformaldehyde for 48 h. After that, it was dehydrated under different concentrations of ethanol, then replaced with xylene, then soaked in wax, embedded and sliced. Finally, Masson and Hematoxylin-Eosin staining was stained on the tissue sections. The optical microscope (Leica DMI4000B) was used to observe the stained images.

### Western blotting

After being treated for 15 days, the protein was extracted from skin tissue by protein extraction kit. The protein sample was separated by 10% page and transferred to the solid carrier for blocking. After blocking, the solid carrier was mixed with primary antibodies against SMA (19245), EGFR (2085), FGFR (9740), AKT (9272), p-AKT (4060), CD31 (3528), VEGF (9698), TNF-α(8184), IL-6 (12912), IL-1β(12703), HO-1 (86806), NQO1 (3187), Nrf2(12721), GAPDH (2118) and Actin (3700) overnight at 4°C, and then incubated with secondary antibodies. All antibodies were purchased from Cell Signaling Technology. The signals were detected by Super ECL Detection Reagent (Tanon, China).

### Immunofluorescence

Firstly, the slices were punched and blocked. Subsequently, the slices were incubated overnight with primary anti-SMA antibody. Next day, SMA were removed and the samples were mixed with the appropriate secondary antibody for 2 h. Finally, the slices were incubated with DAPI for 15 min.

### Statistical analysis

Data were expressed as means ± standard deviations (SD). All statistical analyses were analyzed using SPSS 22.0 (IBM, United States), Graph Pad Prism 6.0 (CA, United States) software. One-way analysis of variance was used to analyze significant differences between multiple groups, and Tukey’s multiple comparisons post-hoc test was followed.

## Results and discussion

### Preparation and characterization of ZnO-Ber/H

**FIGURE 1:**
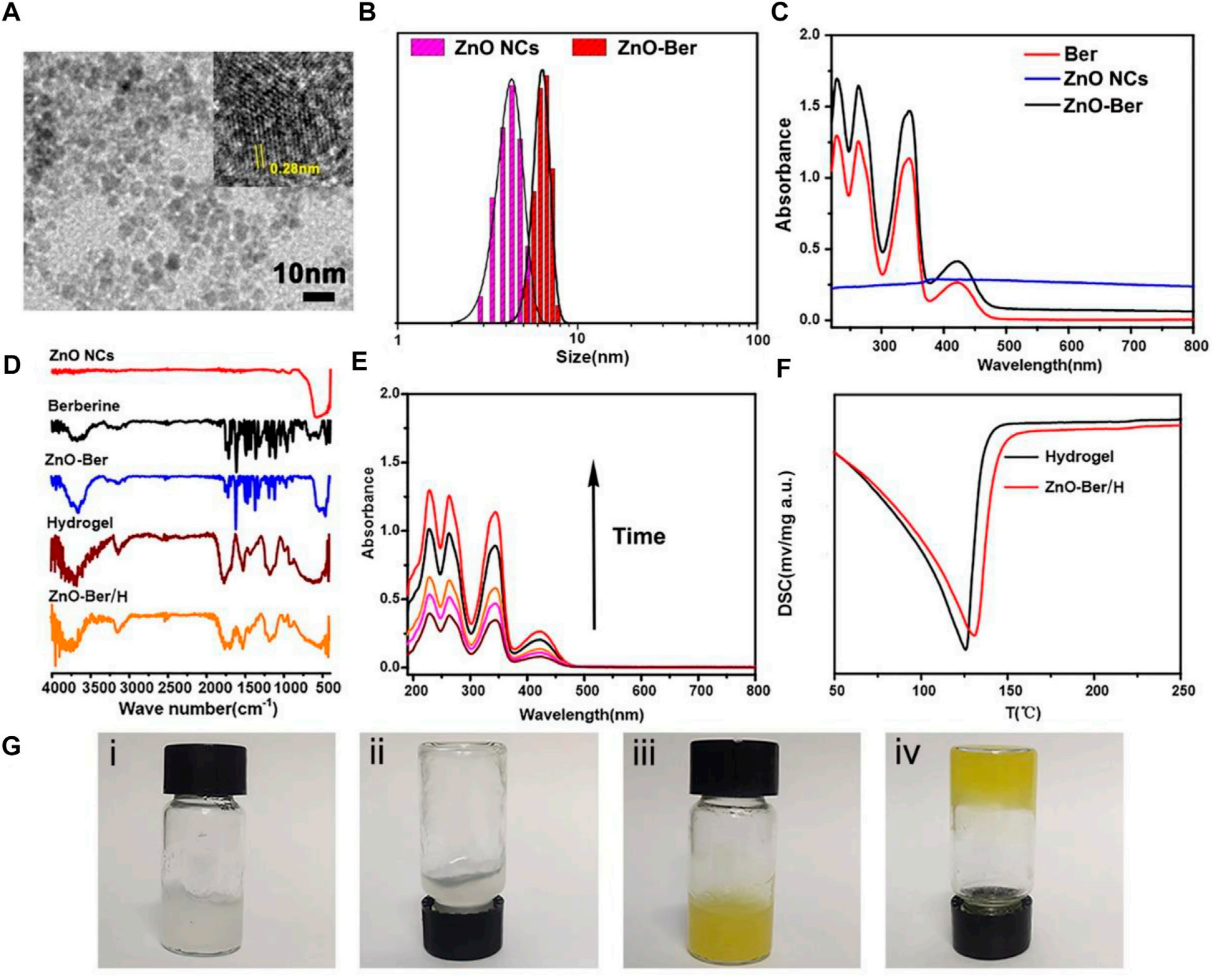
**(A)** TEM images of ZnO NCs, the insert was the labeled distance of 2.8 Å corresponded to the {10 $\bar{1}$ 0} lattice spacing. **(B)** The size and size distributions of ZnO NCs and ZnO-Ber were determined by DLS. **(C–D)** UV–vis data and FTIR spectra data of ZnO NCs, Berberine and ZnO-Ber/H. **(E)** Released of berberine from ZnO-Ber in different time. **(F)** DSC results of the Hydrogel and ZnO-Ber/H. **(G)** Digital photos of Hydrogel and ZnO-Ber/H. Scale bar, 10 nm.

The preparation of ZnO-Ber was carried out by a simple method. At the same time, further characterization was carried out by TEM, UV-visible photometry and FT-IR. Figure 1A was a lattice-resolved image of a single ZnO-Ber seed on the TEM window. The labeled lattice directions corresponded to the perpendicular {10 $\bar{1}$ 0} and {11 $\bar{2}$ 0} planes of ZnO-Ber, confirming that the c-axis was normal to the membrane surface. The TEM images showed that the ZnO-Ber is uniform in size and 5–10 nm in diameter. Particle size data (Figure 1B) indicated that the average particle sizes of successfully prepared ZnO NCs and ZnO-Ber were about 5 and 7 nm. For ZnO NCs, smaller size had better performance. For example, smaller size had higher specific surface area, higher defect concentration, better sterilization (Guy et al., 2019).

In order to verify whether berberine was successfully coated on the surface of ZnO, we tested by UV-vis and FITR. In Figure 1C, we could observe characteristic absorption peaks appearing at 228, 263, 345 and 422 cm^−1^ belonging to berberine in the ZnO-Ber group. At the same time, a characteristic peak at 334 nm was also observed, which belonged to ZnO. The FTIR spectrum results (Figure 1D) showed that the characteristic peaks of benzene ring appeared at 1506 cm^−1^ and 1601 cm^−1^, which was consistent with the benzene ring in berberine. At the same time, it could also be observed around 590 cm^−1^ peak, this peak belonged to the stretching vibration peak of Zn-O. The above FTIR and UV-vis spectral results were sufficient to prove that the synthesis of ZnO-Ber/H was successful.

In wound healing dressings, the performance of sustained release is very important. Subsequently, in Figure 1E, we employed UV spectrophotometry to assess the *in vitro* release of berberine from ZnO-Ber/H. Over time, the UV absorption peak intensity of berberine increased steadily, indicating that ZnO-Ber/H could release berberine stably. Figure 1F presented the DSC graphs. The results indicated that the exothermic peaks of water for Hydrogel and ZnO-Ber/H had gradually moved from 125°C to about 150°C. This indicated that ZnO-Ber/H had stronger moisturizing properties than pure hydrogel, which could be due to the stronger interaction between water and ZnO-Ber/H hydrogel bonds. In Figure 1G, we demonstrated the good fluidity of pure hydrogels (i-ii). After adding ZnO-Ber to the hydrogel (iii-iv), we saw a gel phenomenon that was different from pure hydrogel, which showed that ZnO-Ber/H had great hydrogel properties. These characteristics were crucial in accelerating wound healing.

### ZnO-Ber/H promoted the proliferation and migration of epidermal cells and fibroblasts

**FIGURE 2:**
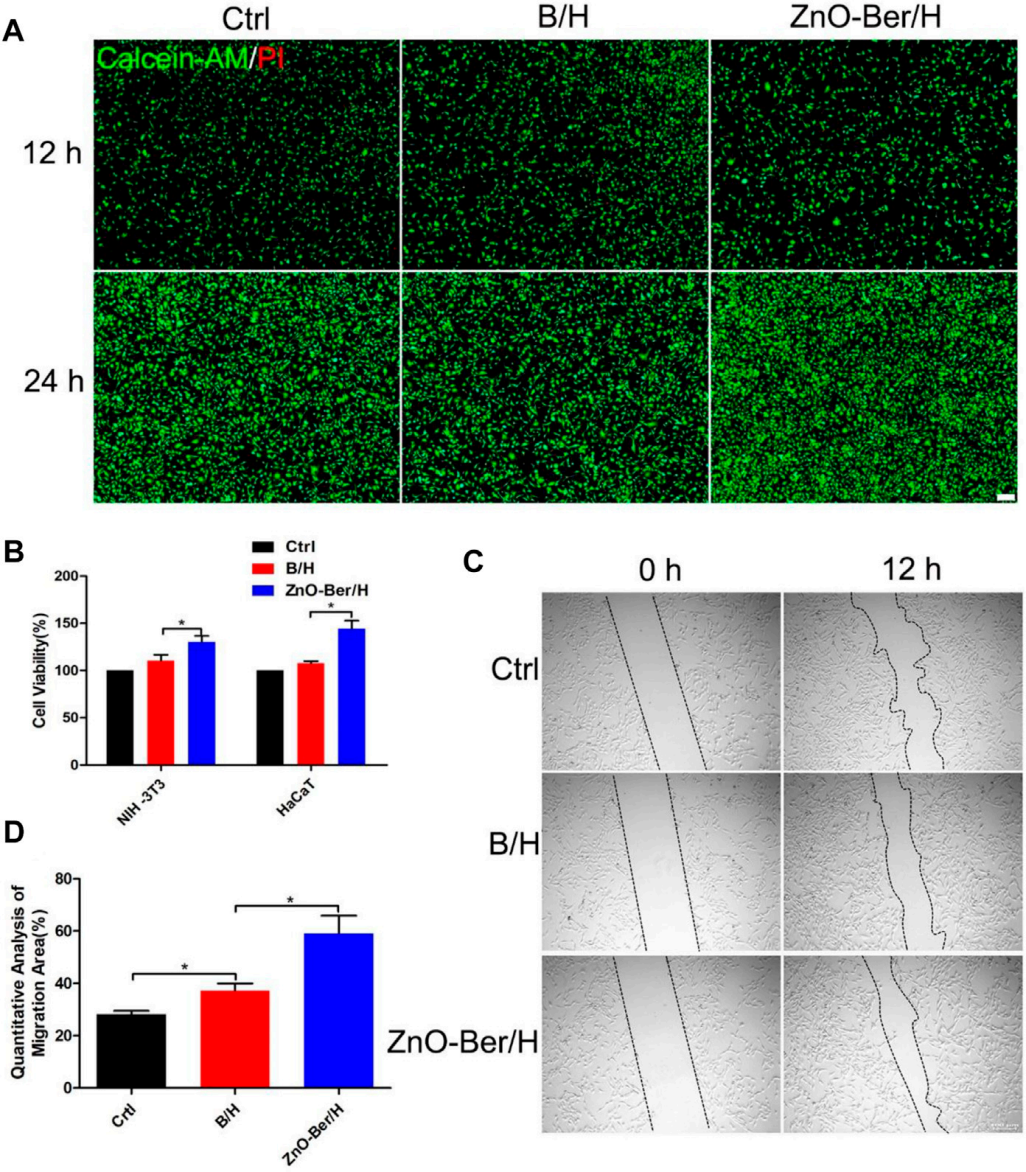
Biocompatibility of the ZnO-Ber/H. **(A)** The cytotoxicity of the Control, B/H and ZnO-Ber/H was determined by live and dead staining at 12 and 24 h **(B)** CCK-8 analysis in control groups, B/H group and ZnO-Ber/H group. **(C)** The scratch-wound assay in control groups, B/H group and ZnO-Ber/H group. **(D)** Migration rates analysis. Statistical analysis: *p ≤ 0.05, **p ≤ 0.01 (Scale bar:100 μm).

During the development of wound healing, the interaction between cells was mainly the interaction between keratinocytes and fibroblasts. With gradual change of wound microenvironment, it was more conducive to take shape of granulation tissue. They were related to wound contraction and collagen production, and they acted a vital role in wound healing (Werne et al., 2007; Watt and Fujiwara, 2011; Li et al., 2018). In Figure 2A, we had proved that our synthesized ZnO-Ber/H had no cytotoxicity and the cells could survive normally without causing cell death through a live-dead staining kit. Figure 2B showed the CCK-8 detection kit proved that ZnO-Ber/H could promote the proliferation of NIH-3T3 and HaCaT.

**FIGURE 3:**
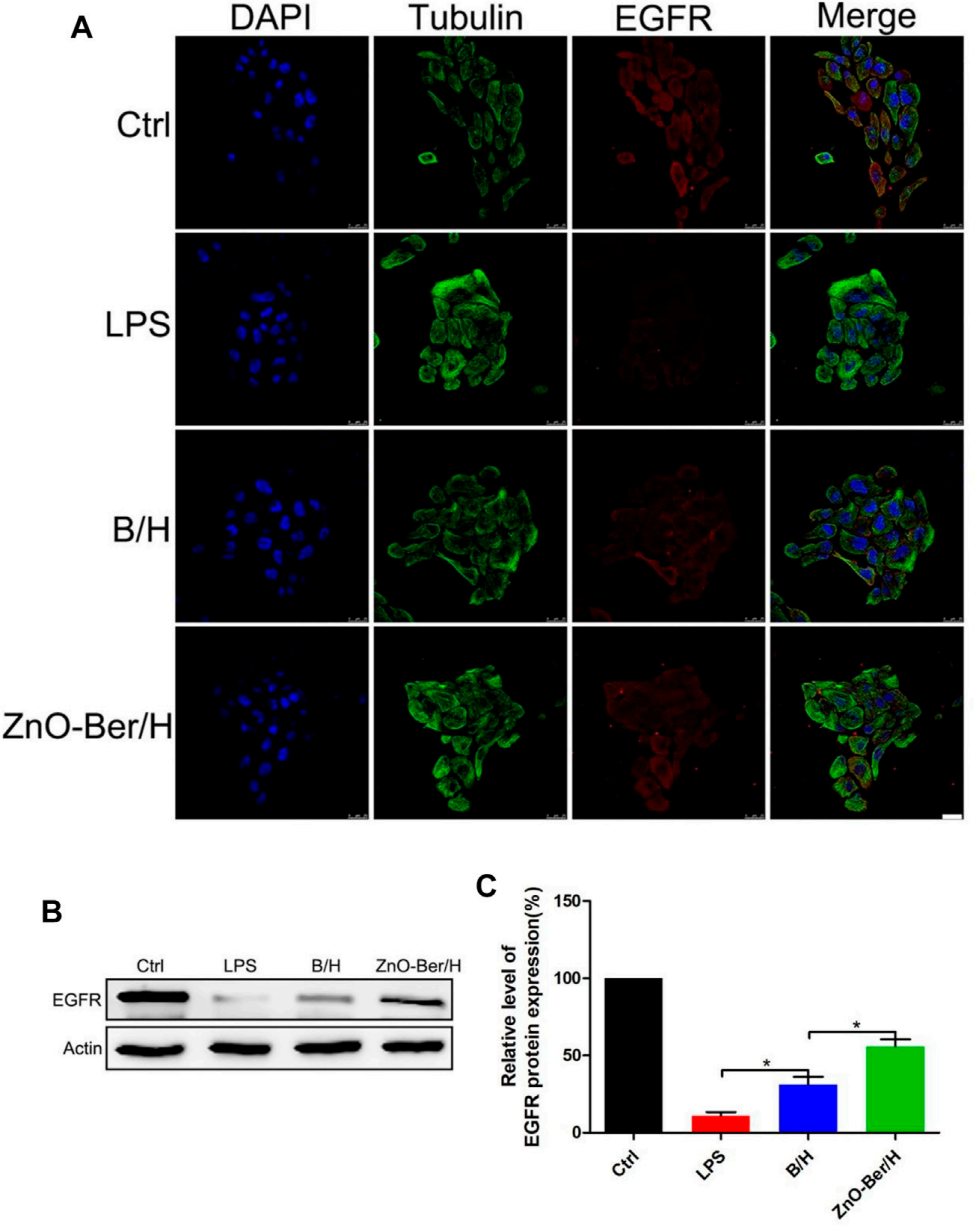
Protein expression of EGFR in HaCaT by Immunofluorescence staining and Western Blot. **(A)** Immunofluorescence staining of EGFR expression of HaCaT cells. **(B)** Western blot detection of expression of EGFR proteins in HaCaT. **(C)** The expression analysis of EGFR. Scale bar: 25 μm. Statistical analysis: *p ≤ 0.05.

In order to study whether it would affect the migration ability of NIH-3T3 cells, cell scratch test was used. In Figures 2C,D, after 12 h of adding ZnO-Ber/H, the mobility of the ZnO-Ber/H group was greater than that of the B/H group, and of course, greater than that of the control group. The mobility of 62.1% in ZnO-Ber/H was better than that in B/H (47.2%) and Ctrl (28.1%) (Figure 2D). It could be proved that ZnO-Ber/H could improve the migration ability of NIH-3T3 cells. Keratinocytes were vital for formation of new epidermis (Kim et al., 2020). EGF/EGFR signaling pathway was one of the most characteristic signaling pathways. They were involved in many stages of wound healing (Repertinger et al., 2004; Lee et al., 2018). Therefore, we firstly observed the expression of EGFR protein on HaCaT. In Figure 3, we pretreated cells with 1 μg/ml LPS, then intervened with ZnO-Ber/H, and finally verified with immunofluorescence and Western blot. Figure 3A showed that after treating cells with LPS, the expression of EGFR protein decreased, while after adding ZnO-Ber/H, the expression of EGFR increased. Accordingly, in Figures 3B,C, we got the same trend. In the later part of the manuscript, we also verified it *in vivo*.

### ZnO-Ber/H could reduce the oxidative stress of fibroblast

**FIGURE 4:**
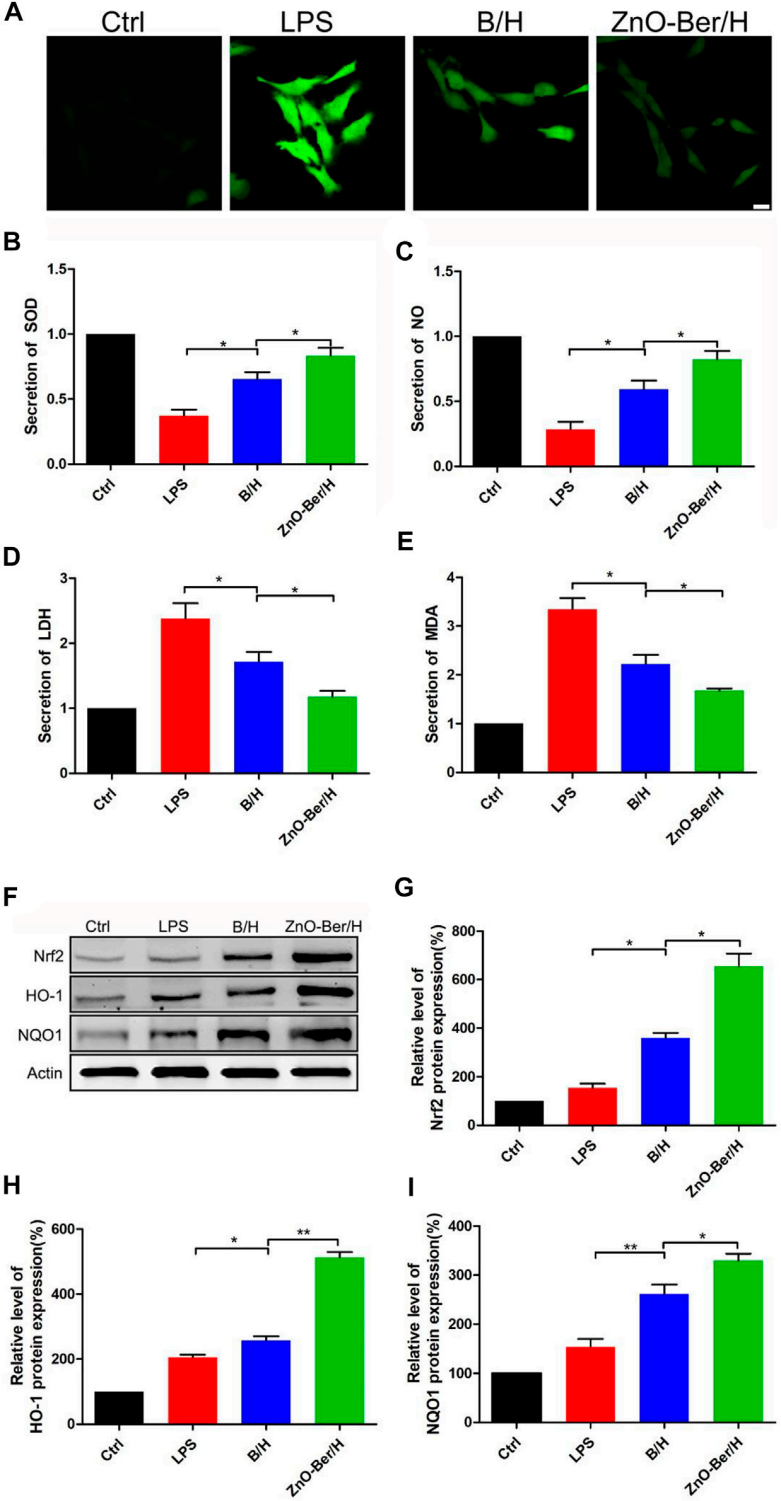
ZnO-Ber/H regulated reactive oxygen production induced by DCFH-DA. Nrf2/HO-1/NQO1 signal pathway participated in the process of ZnO-Ber/H combating oxidative damage. **(A)** Confocal fluorescence microscope examinations of intracellular ROS levels of NIH-3T3. **(B)** Supernatant detections of SOD from NIH-3T3. **(C)** Evaluation of NO secretion from NIH-3T3. **(D)** Supernatant detections of LDH from NIH-3T3. **(E)** Supernatant detections of MDA from NIH-3T3. **(F)** Western blot detection of expression of Nrf2, HO-1, and NQO1 proteins in NIH-3T3. **(G–I)** The relative expression analysis of western blot data. Scale bar: 25 μm. Statistical analysis: *p ≤ 0.05, **p ≤ 0.01.

It had been reported that the accumulation of reactive oxygen species was one of the important reasons for cytotoxicity and delayed wound healing. The removal of reactive oxygen species was more important in the healing process of diabetic chronic wounds (Jiang et al., 2011; Bilgen et al., 2019). In this work, we pretreated fibroblasts with LPS for 1 h, after that, treated the fibroblasts with ZnO-Ber/H. As shown in Figure 4A, compared with group B/H, ZnO-Ber/H could further reduce the intensity of cellular reactive oxygen species. As shown in Figures 4F–I, the expression of Nrf2, HO-1, and NQO1 in ZnO-Ber/H was significantly higher than that of all other cell samples. Next, we used the detection kit to detect SOD, NO, LDH, and MDA. All these factors were related to oxidative stress. SOD is regarded as the most vital enzymes against ROS. MDA could aggravate the damage of cell membrane as the most important product of cell lipid oxidation that reflects the extent of damage of membrane system. NO can reduce the damage of hydroxyl radicals to cells through reacting with hydroxyl radicals, but high concentration of NO can inhibit the synthesis of protein and mitochondrial respiration, which in turn causes apoptosis and necrosis in hepatocytes. LDH is strongly associated with cell death following oxidative stress. LDH levels are measured to determine cytotoxicity. The results were shown in Figures 4B–E, which further verified our guess that the ZnO-Ber/H group could indeed reduce the degree of oxidative stress.

### ZnO-Ber/H could accelerate wound healing

**FIGURE 5:**
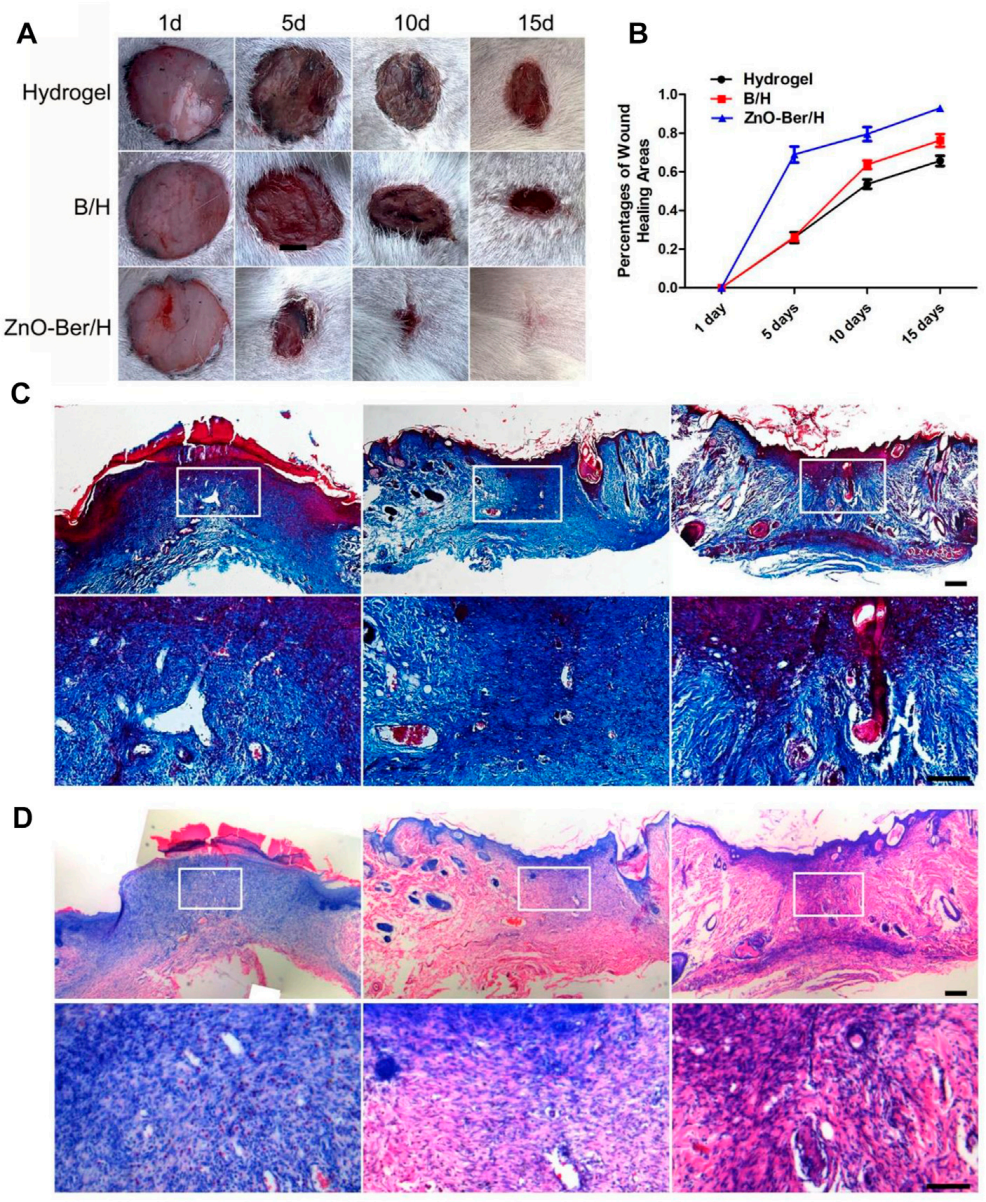
Histological staining of wound site. **(A)** General images of wounds after operation. **(B)** Wound closure rate analysis. **(C–D)** Masson and H&E staining and the partial magnification of Masson and H&E staining. Scale bar: 100 μm.

As shown in Figures 5A,B, all groups were combined for healing evaluation experiments *in vivo*. Compared with the hydrogel group, ZnO-Ber/H significantly improved the wound healing speed. On the 15th day, the percentage of wound contraction in each group was calculated. The percentages of wound shrinkage in the hydrogel, B/H, and ZnO-Ber/H groups were 65.6%, 76.2%, and 92.9%, respectively. The ZnO-Ber/H group had the best wound closure effect (*p* < 0.05). These results demonstrated that ZnO-Ber/H was expected to be a more effective dressing.

Masson and H&E staining indicated the process of tissue collagen formation and re-epithelialization. We need to observe whether the wound has granulation tissue regeneration and wound vascularization 15 days after the injured. The treatment group of ZnO-Ber/H was significantly rich in granulation tissue and vascularization, and new epithelial tissue was formed (Figures 5C,D).

### ZnO-Ber/H promoted the expression of epidermal and vascular related factors

**FIGURE 6:**
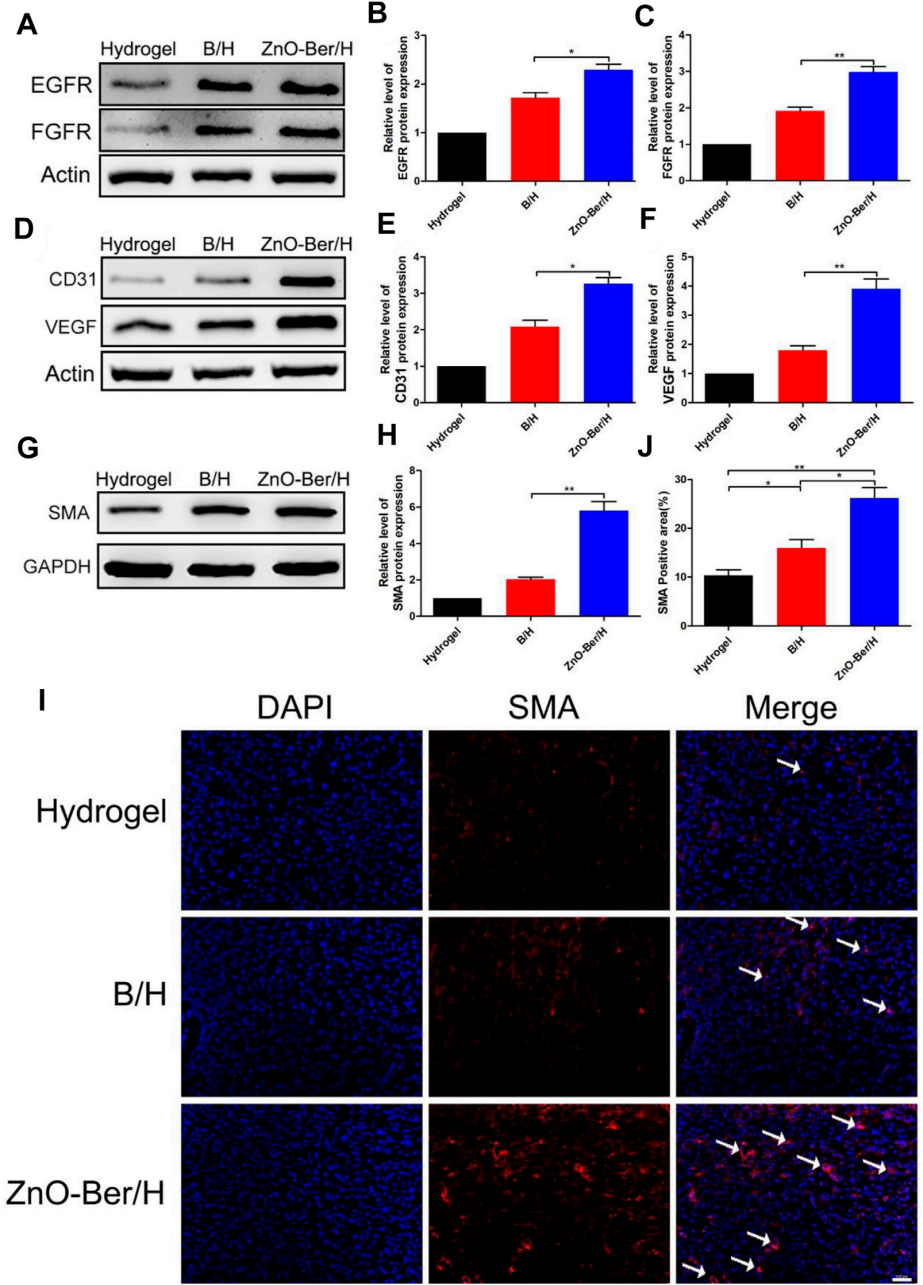
Protein expression of EGFR, FGFR, SMA and AKT/p-AKT in wound healing. **(A)** Western blot expression of EGFR and FGFR proteins. **(B,C)** Western blot expression analysis of EGFR and FGFR. **(D)** Western blot expression of CD31 and VEGF proteins. **(E,F)** Western blot expression analysis of CD31 and VEGF. **(G)** Western blot detection of expression of SMA protein. **(H)** Western blot expression analysis of SMA. **(I)** Immunofluorescence of SMA expression. **(J)** The relative expression analysis of SMA. Statistical analysis: *p ≤ 0.05, **p ≤ 0.01. Scale bar: 100 μm.

As we all known, EGFR was a G protein-coupled receptor on the cell surface. Various cytokine receptors could activate EGFR and promote cell survival, migration and proliferation (Pastore et al., 2008; Shi et al., 2019). Fibroblast growth factors could induce cell proliferation in damaged area of the skin, promoted the production of cytokines and other growth factors, and induced macrophages and monocytes to migrate to the damaged area to remove damaged or dead cells. In addition, FGF also down-regulated the expression of type I procollagen, inhibited the production and deposition of collagen in fibroblasts, thereby prevented the formation of scars (Matuszewska et al., 1994; Mellin et al., 1995; Zhang and Issekutz, 2002; Xie et al., 2011). In this work, we observed the expression of EGFR protein and FGFR protein. We extracted the proteins from the wound tissues of each group, and finally verified them by Western blot. In Figures 6A–C, ZnO-Ber/H group had higher protein expression, indicating that ZnO-Ber/H could promote the expression of EGFR and FGFR proteins in wound healing.

Angiogenesis played an essential role in wound healing. We detected the vascular-associated proteins VEGF and CD31. As shown in Figures 6D–F, the protein of VEGF and CD31 in the ZnO-Ber/H group was higher than other groups. Shrinking microfilament bundles formed by fibroblasts during wound healing were essential for wound contraction (Desmoulière et al., 2005; Li and Wang, 2011). The expression of smooth muscle actin (SMA) could identify fully differentiate myofibroblasts (Ghassemifar et al., 1997). In Figures 6G,H, the expression of SMA in ZnO-Ber/H group was significantly higher than others, indicating that ZnO-Ber/H could shrink the wound more effectively and accelerate wound healing. Meanwhile, we saw the same trend about SMA protein by immunofluorescence in Figures 6I,J.

### ZnO-Ber/H could anti-inflammatory and anti-oxidant stress

**FIGURE 7:**
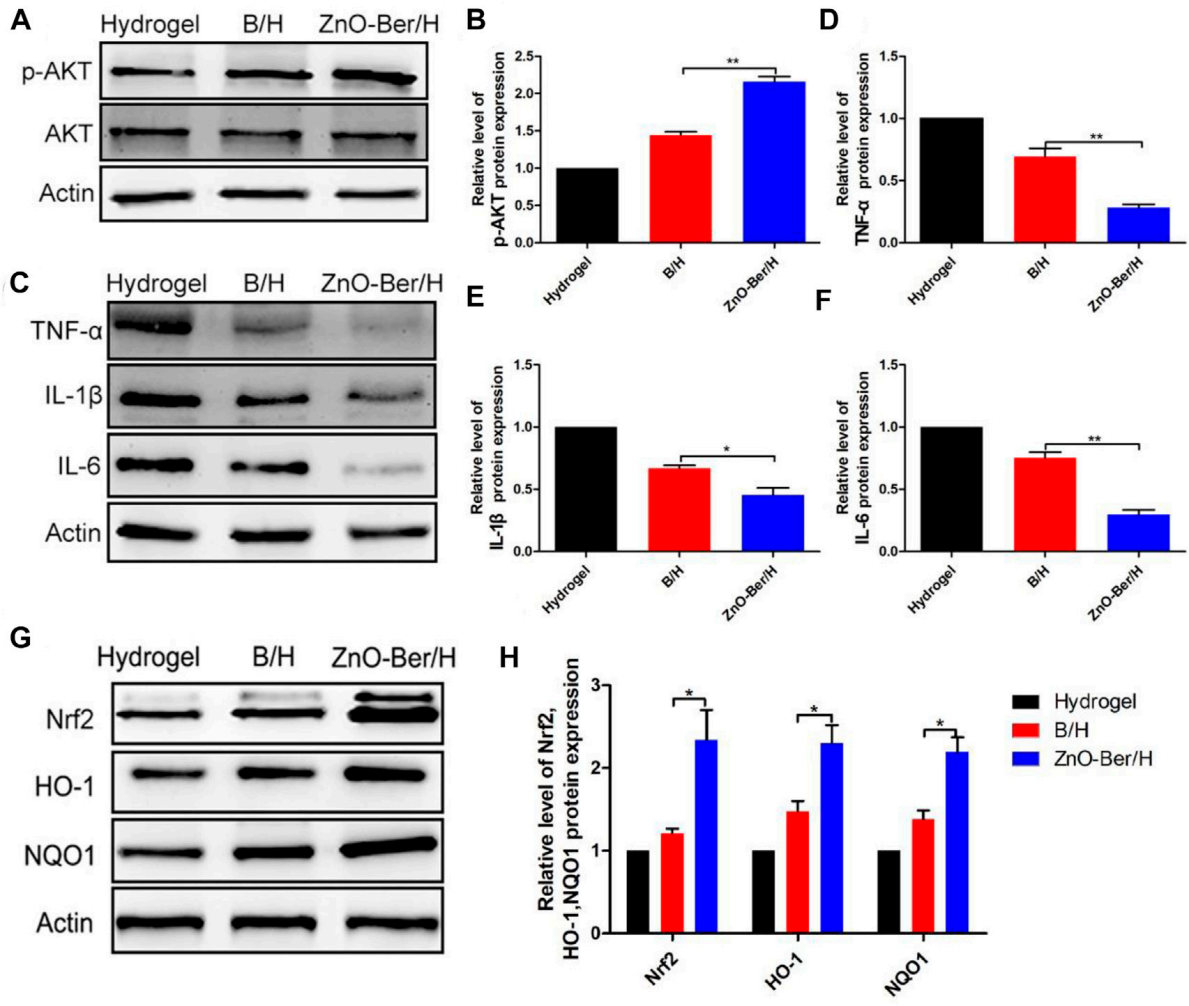
Protein expression of Inflammatory and anti-oxidant stress related factors. **(A)** Western blot detection of expression of total AKT and p-AKT proteins. **(B)** The relative expression analysis of total AKT and p-AKT. **(C)** Western blot expression of Nrf2, HO-1 and NQO1 proteins. **(D–F)** The relative expression analysis of Nrf2, HO-1 and NQO1. **(G)** Western blot expression of TNF-α, IL-1β and IL-6 proteins. **(H)** The relative expression analysis of TNF-α, IL-1β and IL-6. Statistical analysis: *p ≤ 0.05, **p ≤ 0.01.

It had been reported that AKT could phosphorylate the serine site of Nrf2 protein and lead to Nrf2 activation, so as to improve its expression level (Wen et al., 2018). Crosstalk between AKT and Nrf2 signaling pathways could protect cells from inflammation and oxidative damage (Padiya et al., 2014). As mentioned above, berberine had a strong anti-oxidative stress effect (Dodero et al., 2020). One of the most important reasons for chronic refractory wounds caused by diabetes was severe inflammatory infiltration (Samadian et al., 2020). In Figure 7, we tested the content of AKT/p-AKT on the animal model. As shown in Figures 7A,B, after adding ZnO-Ber/H, the content of AKT/p-AKT at the wound increased. In terms of inflammatory factors (Figures 7C–F), after adding ZnO-Ber/H, the expression of TNF-α, IL-1β, and IL-6 were obviously reduced. As shown in Figures 7G,H, compared to others, ZnO-Ber/H groups had obvious enhancement of Nrf2, HO-1 and NQO1. Therefore, we speculated that ZnO-Ber/H might anti-oxidative stress *via* activation of AKT pathway. There had a great relationship with Berberine’s strong anti-inflammatory ability and anti-oxidative stress ability, and had a great relationship with the antibacterial and anti-inflammatory ability of Zn^2+^. Firstly, covered the wound with ZnO-Ber/H to isolate the outside world and created a sterile microenvironment. Then, ZnO-Ber/H activated AKT signaling pathway, and then affected the expression of oxidative stress related factors and inflammation related factors. The changes of these factors would further improve the wound microenvironment and make full preparations for wound healing.

## Conclusion

In conclusion, we proposed a new strategy to address the difficulty in wound healing in diabetic rats. This study was the first to demonstrate the combined ability of ZnO and berberine to successfully synthesize a hydrogel system that effectively treated skin damage in diabetic rats. Using zinc nitrate and berberine as raw materials, through an improved method, a ZnO-Ber/H with moisturizing, anti-inflammatory and antioxidant properties could be directly obtained. This hydrogel could achieve both anti-inflammatory and anti-oxidative effects. Biological applications have broad application prospects. *In vitro*, cell live and dead and migration experiments showed that ZnO-Ber/H has excellent biocompatibility and the ability to induce fibroblast migration. *In vivo* experiments indicated that ZnO-Ber/H could effectively improve the wound healing rate. Masson and H&E staining showed that granulation tissue and blood vessel formation are significantly enriched in the ZnO-Ber/H treated group, and new epithelial tissue is formed. Western bolt and immunofluorescence confirmed that ZnO-Ber/H could enhance the expression of anti-oxidative stress, as well as the expression of anti-inflammatory related factors, thereby significantly promoting granulation formation of tissue and new blood vessels. At the same time, the hydrogel could promote the expression of EGFR and FGFR, thereby affecting the generation of new epithelial tissue. Based on extensive characterization and biological evaluation, ZnO-Ber/H is expected to be a potential candidate material for promoting diabetic wound healing.

## Data Availability

All relevant data is contained within the article: The original contributions presented in the study are included in the article/Supplementary Material, further inquiries can be directed to the corresponding authors.
